# Incorporation of dried blood alpha fetoprotein into traditional first trimester Down syndrome screening service

**DOI:** 10.1002/pd.4596

**Published:** 2015-05-13

**Authors:** Jonathan Carmichael, David Krantz, Hsiao-Pin Liu, David Janik, Terrence Hallahan

**Affiliations:** 1 PerkinElmer LaboratoriesMelville, NY, USA

## Abstract

**Abstract:**

**Objective**

The aim of this study was to determine whether incorporation of dried blood alpha fetoprotein (AFP) into first trimester screening using the biochemical markers free Beta human chorionic gonadotropin (hCG) and pregnancy-associated plasma protein A (PAPP-A) can improve screening performance.

**Methods**

A retrospective study of 34 Down syndrome and 1185 unaffected dried blood specimens. First trimester dried blood AFP was performed using in-house immunofluorometric time-resolved assay. False positive and detection rates were determined from modeling.

**Results**

The multiple of the median in Down syndrome cases was 0.73. At a fixed 5% false positive rate, incorporating AFP into a free Beta hCG, PAPP-A, and nuchal translucency protocol adds 2% detection resulting in detection rates of 92% to 94% depending on the gestational age of the blood draw. At a fixed 90% detection rate, AFP reduced the false positive rate by 1.0 to 1.6 percentage points depending on gestational age. Using a cutoff of 1/1000, the combination of free beta hCG, PAPP-A, AFP, and nuchal translucency achieved a detection rate of 96% with a false positive rate of 8.4% to 9.9%. Adding in nasal bone increased detection to 98% while reducing false positive rates to 4.1% to 4.7%.

**Conclusion**

Inclusion of dried blood AFP into traditional first trimester screening improves detection while optimizing contingent protocols so that cell-free fetal DNA testing may be offered in a more cost effective manner. © 2015 The Authors. *Prenatal Diagnosis* published by John Wiley & Sons Ltd.

**What’s already known about this topic?:**

1. Traditional first trimester Down syndrome screening programs can achieve detection rates of 90% for a 5% false positive rate.
2. Cell-free fetal DNA screening can achieve high detection rates and low false positive rates but due to its cost may be ill-suited to population-wide screening.

**What does this study add?:**

1. Incorporation of AFP into a dried blood first trimester screening program utilizing free beta hCG and PAPP-A can improve detection rates and/or false positive rates.
2. Improved performance of first trimester screening with AFP can optimize contingent protocols so that cffDNA testing may be offered in a more cost effective manner.

## Introduction

Maternal serum Down syndrome screening was initially introduced as an adjunct to second trimester open neural tube defect screening using alpha fetoprotein (AFP).^1^,^2^ Although the initiation of Down syndrome screening represented a significant advance in prenatal care, detection rates were poor. Efforts were made to improve second trimester screening, and eventually, multiple serum analyte marker protocols were developed bringing detection rates up to 60% to 70%.^3^

Further improvements to the screening process were achieved when screening moved into the first trimester where a combination of free beta human chorionic gonadotropin (hCG), pregnancy-associated plasma protein A (PAPP-A), and the ultrasound marker nuchal translucency (NT) has been shown to achieve a detection rate of 90% at a 5% false positive rate.^4^–^7^ Even higher detection efficiency was observed with the inclusion of nasal bone.^8^ First trimester screening had the advantage of not only higher detection rates but also provided pregnant women, with an affected fetus, more time to make decisions about their pregnancy prior to when they were obviously pregnant. However, during the transition from second to first trimester screening, AFP was not included in most screening programs.

A number of studies published in the 1980s showed that first trimester AFP levels are reduced in cases of Down syndrome.^9^–^12^ A meta-analysis was performed in 1999 encompassing 26 studies and 542 cases showing a geometric mean in Down syndrome pregnancies of 0.79 multiples of median (MoM).^13^ More recently, Nicolaides *et al*.^14^ observed the first trimester AFP median MoM to be 0.78 in 65 cases of Down syndrome. The authors found that inclusion of AFP into the standard first trimester screening protocol reduced the false positive rate 10% to 15% depending on the risk cutoff.

In 1982, Mizejewski demonstrated the feasibility of measuring AFP in dried blood, albeit in newborns.^15^ In the early 1990s, we implemented a second trimester dried blood free beta hCG/AFP assay^16^ and later implemented a first trimester Down syndrome screening program with a dual-analyte dried blood spot free Beta hCG/PAPP-A assay.^7^

More recently, advances in technology have led to the implementation of Down syndrome screening with cell-free fetal DNA (cffDNA). cffFDNA screening can provide very high detection rates with very low false positive rates.^17^–^19^ Although cffDNA screening holds great promise, it may be ill-suited for population-wide screening because of its high cost and resulting large financial burden on national healthcare systems and private individuals. In addition, failure rates of 1% to 5% indicate that the reported detection rates may be higher than the actual detection rates that can be achieved in practice.^20^ Finally, maternal serum screening markers can identify patients at risk for other quite common adverse outcomes such as preeclampsia, intrauterine growth restriction, and preterm delivery.^21^ Several authors have suggested a contingent strategy for Down syndrome screening in which patients are initially provided traditional screening, and only those who are identified as at increased risk are followed up with cffDNA testing.^22^–^25^ A contingent strategy may be used with standard cutoff risks, but it may also be used with lower risk cutoffs such as 1/1000 to maximize detection efficiency.

We sought to evaluate the possibility of improved screening performance as the first step in a cfDNA contingent strategy by evaluating dried blood AFP in addition to free beta hCG and PAPP-A.

## Materials and Methods

A retrospective analysis was performed on dried blood specimens from 34 Down syndrome pregnancies and 1185 unaffected controls. The study was approved by an external institutional review board (New England IRB). The Down syndrome cases were the remaining available blood spots from a previously described sample set.^7^ Unaffected controls were from specimens submitted to our laboratory and stored at −20 °C for up to 3 years. The average gestational age was 83.6 days [standard deviation (SD): 7.98], and the average maternal age was 31.0 years (SD: 5.00) in the unaffected group. The population was 72.7% Caucasian, 9.0% Hispanic, 5.7% African American, 3.9% Asian, and 8.7% other ethnic groups. In the Down syndrome group, the average gestational age was 83.3 days (SD: 6.02), and the average maternal age was 37.5 years (SD: 3.92). Among the Down syndrome group, 73.5% were Caucasian, 8.8% Asian, 8.8% Hispanic, 2.9% African American, and 6.0% other ethnic groups.

The previously determined free beta hCG and PAPP-A MoM values for Down syndrome pregnancies were used.^7^ For unaffected patients, free beta hCG and PAPP-A MoM values from routine screening were used. Samples were analyzed for AFP with a laboratory-developed automated time-resolved fluorometric assay performed on the AutoDELFIA instrument. The assay utilizes a dried blood spot direct punch ‘sandwich-type’ immunoassay. A monoclonal antibody directed against AFP is immobilized to a microplate well. During the elution of the dried blood spot, AFP binds to the coated wells and interacts with europium labeled monoclonal antibodies directed at a different antigenic site. The addition of enhancement solution causes dissociation of the europium from the antibody resulting in highly fluorescent chelates. The resulting fluorescence is proportional to the concentration of AFP in the sample. Gestational age specific medians were determined using the subgroup of Caucasian patients, and MoM values were determined. MoMs were then adjusted for weight and ethnicity.

Parameters for log-Gaussian distributions were calculated as follows: (1) the free beta hCG and PAPP-A parameters and the correlation of free beta and PAPP-A for the unaffected and Down syndrome distributions were taken from the previously published study.^7^ (2) For AFP, the mean was based on the log of the median MoM value in Down syndrome cases and set equal to 0 for unaffected cases. (3) The SD was determined by subtracting the 10th percentile from the 90th percentile on a log scale and dividing by 2.563. (4) The correlation coefficients for AFP with free Beta and PAPP-A were determined from the Spearman rank correlation coefficient. (5) The correlation between each biochemical marker and NT was set equal to 0. (6) NT distribution parameters were obtained from published data.^26^

False positive and detection rates were determined by simulation based on the Gaussian distribution parameters and the age distribution of live births in the USA in 2012.^27^ MoM values for biochemistry and nuchal translucency were simulated using the parameters of the Gaussian distributions. Nasal bone was factored in by using the likelihood ratio method described by Cicero *et al*.^28^ For each set of simulated MoM values, a simulated nasal bone result of ‘absent’ or ‘present’ was determined using the following method. First, a random number was generated, and if the random number was less than 0.157 (the percent of live births with African American mothers^27^), the ethnicity was considered to be African American. Second, the simulated NT MoM was converted to an NT value. Third, the expected probability of an absent nasal bone value was determined based on crown-rump length (CRL), NT, and ethnicity and outcome (Down syndrome or unaffected). Fourth, another random number was generated, and if the random number was less than the likelihood (probability) of an absent nasal bone, the simulated nasal bone value was considered ‘absent’, otherwise it was considered ‘present’. The likelihood ratio for nasal bone was multiplied by the likelihood ratio from the Gaussian distribution. Risk values were determined by multiplying the likelihood ratio by the *a priori* risk. In all simulations, the ultrasound was considered to be performed at 12 weeks of gestation.

## Results

**Figure 1:**
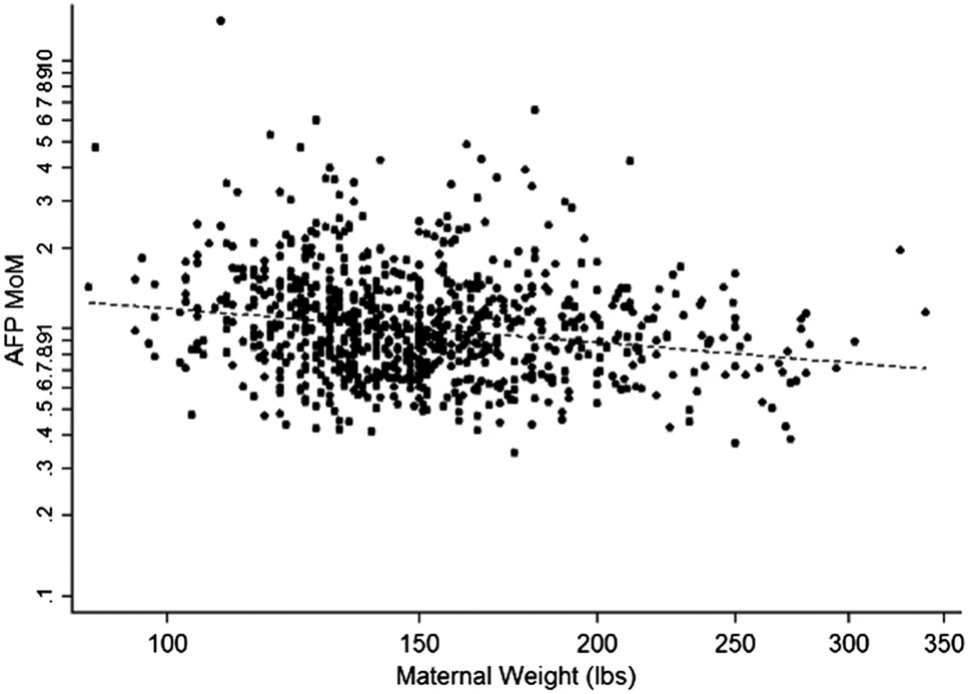
Regression of alpha fetoprotein (AFP) multiple of median (MoM) versus maternal weight. AFP MoM = 10^(−0.4193*log weight + 0.9125). Dashed line indicates regression line

Alpha fetoprotein medians increased by 36.7% per week between 9 and 13 weeks of gestation in control specimens (Table1). Figure1 shows a negative correlation between AFP MoM and maternal weight. Based on the data in Figure1, a weight adjustment equation was developed using the following formula: Expected MoM = 10^(−0.4193*log weight + 0.9125). Based on this formula, a 100-pound (45.35 kg) patient would be expected to have a MoM value of 1.19 and a 200-pound (90.7 kg) patient would be expected to have a MoM value of 0.89. After weight adjustment, the median MoM was 1.00, 1.11, 0.95, 0.88, and 0.97 in the Caucasian, African American/Caribbean, Hispanic, Asian, and other ethnic groups, respectively. Only the African American/Caribbean ethnic group was statistically significantly different than the Caucasian group (*P* = 0.02, Wilcoxon rank-sum test), and an ethnic adjustment was incorporated for this group.

**Table 1:** Observed and regressed medians for AFP in Caucasian unaffected patients

			Median AFP (IU/mL)	
Gawk	*N*	Median day	Observed	Regressed^a^
9	112	67	9.95	8.06
10	117	74	12.23	11.02
11	157	82	19.04	15.06
12	289	87	24.32	20.59
13	178	92	28.43	28.14

AFP, alpha fetoprotein.

Regression formula: AFP = 10^[−0.31585 + 0.13579[Table-fn tf1-1](gestational age in weeks)].

At completed weeks.

The median MoM in Down syndrome cases was 0.73. There was not a statistically significant increase in the median MoM values in Down syndrome with gestational age. The SD of AFP log10(MoM) values was 0.1838 and 0.1784 in Down syndrome and unaffected pregnancies, respectively. In Down syndrome, the correlation of AFP with free beta hCG and PAPP-A was 0.24864 and −0.06557, respectively. In unaffected pregnancies, the correlation of AFP with free Beta hCG and PAPP-A was 0.04079 and 0.04254, respectively.

Univariately, at a fixed 5% false positive rate, free Beta hCG, PAPP-A, and AFP with maternal age detected 48%, 51%, and 40%, respectively. At a fixed 5% false positive rate, including AFP into the combination of free beta HCG, PAPP-A, and nuchal translucency added 2% detection regardless of gestational age at blood draw. The addition of nasal bone added a further 4 to 6 percentage points to detection at a fixed 5% false positive rate (Table2). At a fixed 2% false positive rate, AFP added 2% to 3% detection depending on the gestational age of the blood draw while including nasal bone added an additional 7 to 9 percentage points (Table2). Depending on gestational age, at a fixed 90% detection rate, AFP reduced the false positive rate by 1.0 to 1.6 percentage points, while inclusion of nasal bone further reduced the false positive rate down to 0.3% to 0.5% (Table3). Similarly, at a fixed 95% detection rate, AFP reduced the false positive rate by 2.6 to 4.2 percentage points and inclusion of nasal bone reduced false positive rates even further down to 1.2% to 1.7%.

**Table 2:** Detection rate at a fixed 2% and 5% false positive rate for protocols including maternal age and given markers based on gestation at blood draw

Gestational age	NT/free beta hCG/PAPP-A (%)	NT/free beta hCG/PAPP-A/AFP (%)	NT/free beta hCG/PAPP-A/AFP/NB (%)
Fixed 2% false positive rate			
9	88	90	97
10	86	89	96
11	85	88	96
12	85	87	96
13	84	87	96
Fixed 5% false positive rate			
9	92	94	98
10	92	94	98
11	91	93	98
12	91	93	98
13	90	92	98

hCG, human chorionic gonadotropin; NT, nuchal translucency; PAPP-A, pregnancy-associated plasma protein A; AFP, alpha fetoprotein; NB, nasal bone.

Ultrasound performed at 12 weeks of gestation.

**Table 3:** False positive rate at a fixed 90% and 95% detection rate for protocols including maternal age and given markers based on gestation at blood draw

Gestational Age	NT/free beta hCG/PAPP-A (%)	NT/free beta hCG/PAPP-A/AFP (%)	NT/free beta hCG/PAPP-A/AFP/NB (%)
Fixed 90% detection rate			
9	3.1	2.1	0.3
10	3.6	2.5	0.4
11	4.2	2.8	0.4
12	4.6	3.1	0.5
13	4.9	3.3	0.5
Fixed 95% detection rate			
9	8.5	5.9	1.2
10	9.8	6.8	1.4
11	11.1	7.6	1.5
12	12.1	8.1	1.6
13	12.7	8.5	1.7

hCG, human chorionic gonadotropin; NT, nuchal translucency; PAPP-A, pregnancy-associated plasma protein A; AFP, alpha fetoprotein.

Ultrasound performed at 12 weeks of gestation.

Table4 shows the false positive rate and detection rate at various risk cutoffs. AFP improves detection and/or reduces the false positive rate for each cutoff and gestational age at blood draw.

**Table 4:** False positive and detection rate at various risk cutoffs for protocols including maternal age and given markers based on gestation at blood draw

	NT/free beta hCG/PAPP-A		NT/free beta hCG/PAPP-A/AFP		NT/free beta hCG/PAPP-A/AFP/NB	
Gestational age (week)	FPR (%)	Detection (%)	FPR (%)	Detection (%)	FPR (%)	Detection (%)
Risk cutoff = 1/10						
9	0.2	71	0.2	73	0.1	85
10	0.2	69	0.2	72	0.1	84
11	0.2	68	0.2	71	0.1	84
12	0.2	67	0.2	70	0.2	83
13	0.2	67	0.2	69	0.2	83
Risk cutoff = 1/300						
9	3.8	91	3.5	93	1.9	96
10	4.0	91	3.7	92	2.0	96
11	4.2	90	3.9	92	2.0	96
12	4.3	90	4.0	91	2.1	96
13	4.3	89	4.0	91	2.1	96
Risk cutoff = 1/1000						
9	9.5	95	8.4	96	4.1	98
10	10.1	95	9.0	96	4.4	98
11	10.6	95	9.4	96	4.5	98
12	11.0	95	9.7	96	4.6	98
13	11.2	94	9.9	96	4.7	98
Risk cutoff = 1/2500						
9	17.3	98	14.8	98	7.1	99
10	18.6	97	15.9	98	7.6	99
11	19.7	97	16.8	98	7.9	99
12	20.5	97	17.3	98	8.1	99
13	21.0	97	17.7	98	8.3	99

hCG, human chorionic gonadotropin; NT, nuchal translucency; PAPP-A, pregnancy-associated plasma protein A; AFP, alpha fetoprotein; FPR, false positive rate.

Ultrasound performed at 12 weeks of gestation.

## Discussion

Our data show that inclusion of AFP into the first trimester Down syndrome screening process improves screening performance. These results are in agreement with recently published data^14^ and a previous meta-analysis.^13^ Previous studies have demonstrated that the combination of free beta hCG and PAPP-A performs better earlier in pregnancy and that feature was incorporated into our model.^7^,^26^,^29^ As a result, our results indicate that screening performance may be further improved when blood is collected prior to ultrasound (Tables4). One study showed that AFP performed better later in pregnancy although 75% of the Down cases were in second trimester.^29^ More data are needed to determine if inclusion of AFP will mitigate the trend in improved performance earlier in pregnancy.

Collection of blood specimens prior to ultrasound may optimize the screening process in patients that provided their final risk result immediately at the conclusion of their ultrasound exam.^30^ As a result, the potential exists for counseling and blood draw for cffDNA screening without further delays in scheduling patients for additional appointments.

A previous study in which our group participated evaluated the cost of hybrid, contingent, and universal cffDNA screening strategies.^25^ A hybrid strategy consists of offering cffDNA to those 35 years and older and those under 35 years old who are at increased risk for Down syndrome.^25^ A contingent strategy consists of offering cffDNA to only those who are at increased risk with traditional screening regardless of age, while a universal strategy consists of offering cffDNA to all patients without traditional screening. That study concluded that a contingent strategy was the most cost effective and that utilization of a 1/1000 cutoff could further reduce costs.

Using the same model but adding in the cost of AFP and the improved false positive and detection rates of traditional screening showed that the cost/patient (including cost of missed cases and follow-up cffDNA testing) would be further reduced in the contingent strategy from $430 per patient to $407 per patient without nasal bone and from $332 to $303 per patient with nasal bone. Using a 1/1000 cutoff with the contingent strategy, inclusion of AFP would result in a reduction in cost from $409 to $388 without nasal bone and from $320 to $295 with nasal bone. Further studies based on the American healthcare system would be helpful in validating these results.

With the advent of cffDNA testing, Down syndrome screening policies are currently influx. Utilization of AFP, which has long been used in second trimester screening, in first trimester screening is a cost-effective addition that can reduce screen positive rates and increase detection rates. This is especially important when the follow-up of screening involves an invasive procedure. However, when the follow up is cffDNA testing, the addition of AFP not only maximizes detection efficiency but also significantly lowers the monetary burden on the healthcare system.

**What’s Already Known About this Topic?**

1. Traditional first trimester Down syndrome screening programs can achieve detection rates of 90% for a 5% false positive rate.
2. Cell-free fetal DNA screening can achieve high detection rates and low false positive rates but due to its cost may be ill-suited to population-wide screening.

**What Does this Study Add?**

1. Incorporation of AFP into a dried blood first trimester screening program utilizing free beta hCG and PAPP-A can improve detection rates and/or false positive rates.
2. Improved performance of first trimester screening with AFP can optimize contingent protocols so that cffDNA testing may be offered in a more cost effective manner.

## References

[b1] Merkatz IR, Nitowsky HM, Macri JN, Johnson WE (1984). An association between low maternal serum alpha-fetoprotein and fetal chromosomal abnormalities. Am J Obstet Gynecol.

[b2] Cuckle HS, Wald NJ, Lindenbaum RH (1984). Maternal serum alpha-fetoprotein measurement: a screening test for Down syndrome. Lancet.

[b3] Tomasi TB (1977). Structure and function of alpha-fetoprotein. Annu Rev Med.

[b4] Krantz DA, Hallahan TW, Orlandi F (2000). First-trimester Down syndrome screening using dried blood biochemistry and nuchal translucency. Obstet Gynecol.

[b5] Nicolaides KH, Spencer K, Avgidou K (2005). Multicenter study of first-trimester screening for trisomy 21 in 75 821 pregnancies: results and estimation of the potential impact of individual risk-orientated two-stage first-trimester screening. Ultrasound Obstet Gynecol.

[b6] Malone FD, Canick JA, Ball RH (2005). First-trimester or second-trimester screening, or both, for Down’s syndrome. N Engl J Med.

[b7] Krantz D, Hallahan T, Ravens R (2011). First trimester Down syndrome screening with dried blood spots using a dual analyte free beta hCG and PAPP-A immunofluorometric assay. Prenat Diagn.

[b8] Cicero S, Curcio P, Papageorghiou A (2001). Absence of nasal bone in fetuses with trisomy 21 at 11–14 weeks of gestation: an observational study. Lancet.

[b9] Brambati B, Simoni G, Bonacchi I, Piceni L (1986). Fetal chromosomal aneuploidies and maternal serum alpha-fetoprotein levels in first trimester. Lancet.

[b10] Barkai G, Shaki R, Pariente C, Goldman B (1987). First trimester alpha fetoprotein levels in normal and chromosomally abnormal pregnancies. Lancet.

[b11] Milunsky A, Wands J, Brambati B (1988). First-trimester maternal serum a-fetoprotein screening for chromosome defects. Am J Obstet Gynecol.

[b12] Cuckle HS, Wald NJ, Barkai G (1988). First-trimester biochemical screening for Down syndrome. Lancet.

[b13] Cuckle HS, van Lith JM (1999). Appropriate biochemical parameters in first-trimester screening for Down syndrome. Prenat Diagn.

[b14] Nicolaides KH, Wright D, Poon LC (2013). First-trimester contingent screening for trisomy 21 by biomarkers and maternal blood cell-free DNA testing. Ultrasound Obstet Gynecol.

[b15] Mizejewski GJ, Bellisario R, Beblowski DW, Carter TP (1982). Commercial radioimmunoassay kit for measurement of alpha-fetoprotein adapted for use with dried blood specimens from newborns. Clin Chem.

[b16] Macri JN, Anderson RW, Krantz DA (1996). Prenatal maternal dried blood screening with alpha-fetoprotein and free beta-human chorionic gonadotropin for open neural tube defect and Down syndrome. Am J Obstet Gynecol.

[b17] Ehrich M, Deciu C, Zwiefelhofer T (2011). Noninvasive detection of fetal trisomy 21 by sequencing of DNA in maternal blood: a study in a clinical setting. Am J Obstet Gynecol.

[b18] Palomaki GE, Kloza EM, Lambert-Messerlian GM (2011). DNA sequencing of maternal plasma to detect Down syndrome: an international clinical validation study. Genet Med.

[b19] Bianchi DW, Platt LD, Goldberg JD (2012). Maternal blood is source to accurately diagnose fetal aneuploidy (MELISSA) study group. Genome-wide fetal aneuploidy detection by maternal plasma DNA sequencing. Obstet Gynecol.

[b20] Langlois S, Brock JA (2013). Current status in non-invasive prenatal detection of Down syndrome, trisomy 18, and trisomy 13 using cell-free DNA in maternal plasma. J Obstet Gynaecol Can.

[b21] Gagnon A, Wilson RD, Audibert F (2008). Obstetrical complications associated with abnormal maternal serum markers analytes. J Obstet Gynaecol Can.

[b22] Cuckle H, Benn P, Pergament E (2013). Maternal cfDNA screening for Down syndrome–a cost sensitivity analysis. Prenat Diagn.

[b23] Okun N, Teitelbaum M, Huang T (2014). The price of performance: a cost and performance analysis of the implementation of cell-free fetal DNA testing for Down syndrome in Ontario. Canada Prenat Diagn.

[b24] Morris S, Karlsen S, Chung N (2014). Model-based analysis of costs and outcomes of non-invasive prenatal testing for Down’s syndrome using cell free fetal DNA in the UK national health service. PLoS One.

[b25] Evans MI, Sonek JD, Hallahan TW, Krantz DA (2015). Cell-free fetal DNA screening in the USA: a cost analysis of screening strategies. Ultrasound Obstet Gynecol.

[b26] Cuckle HS, Benn PA, Milunsky A, Milunky J (2010). Multi-marker maternal serum screening for chromosomal abnormalities. Genetic Disorders and the Fetus: Diagnosis, Prevention and Treatment.

[b27] Centers for Disease Control and Prevention. National Center for Health Statistics http://www.VitalStats./nchs/vitalstats.htm.

[b28] Cicero S, Rembouskos G, Vandecruys H (2004). Likelihood ratio for trisomy 21 in fetuses with absent nasal bone at the 11–14-week scan. Ultrasound Obstet Gynecol.

[b29] Spencer K, Crossley JA, Aitken DA (2002). Temporal changes in maternal serum biochemical markers of trisomy 21 across the first and second trimester of pregnancy. Ann Clin Biochem.

[b30] Fox NS, Rebarber A, Klauser CK (2011). First-trimester aneuploidy risk assessment: the impact of comprehensive counseling and same-day results on patient satisfaction, anxiety, and knowledge. Am J Perinatol.

